# Knowledge on allergic diseases by community health workers

**DOI:** 10.1186/1939-4551-8-S1-A15

**Published:** 2015-04-08

**Authors:** Magna Quadros Coelho, Anna Gruber, Anne Muriel Soares, Enedina Almeida, Isadora Alkmim

**Affiliations:** 1 Unimontes, Brazil

## Background

Although allergy affects thousands of people, economic and social costs with allergic diseases are still neglected. The model of primary health care (PHC) is based on the premise that prevention and health promotion are key. The community health workers (CHWs) are necessarily native community in which it operates, thus having a strong bond with the users of the health service. They are important in the transmission of information and monitoring of the environmental conditions in which patients are entered and verify the correct use of medicines. So, they are professionals who must be trained to assist allergy sufferers and their families, about the health-disease-care and quality of life process. The aim of this study was to strengthen the link between education, health and community services.

## Methods

For evaluated the knowledge about allergic diseases, was made a cross-sectional study with CHWs in Montes Claros, MG, Brazil. A questionnaire was administered for 136 individuals of 33 PHC posts. The questions were about allergic diseases, its origin, treatment and care. This study was approved by the Ethics Committee.

## Results

In 136 CHWs, 71% were female, 21% male and 8% were not identified, and they were aged between 21 to 58 years. As for education, 3% elementary School had Full, 1% had not completed High School, 54% had completed high school, 14% had higher education incomplete, 10% had completed higher education, has 1% technical Level, and 17% not answered. In closed question about knowledge of allergic diseases, 80% of CHWs responded positively; 18% ignored and 2% did not answer. But on the same issue but descriptive, 22% did not answer, and many placements were related to causes and myths and not the specific allergic diseases. Questions on knowledge of treatment and care for allergic diseases were positive for 60% of CHWs and unknown for 40%. When asked about previous participation in training on allergic, disease 18% had a positive response, 80% negative and 2% did not answer. Of CHWs 92% showed interest in participating in a training activity on allergic diseases, and 6% showed no interest and 2% did not answer.

## Conclusions

Based on this study, we found the lack about knowledge of allergic diseases, treatments and care by CHWs. Therefore will be serving as a basis to elaborate a project extension to enable them on this topic.

